# Diagnostic validity of premenstrual dysphoric disorder: revisited

**DOI:** 10.3389/fgwh.2023.1181583

**Published:** 2023-11-27

**Authors:** Shalini S. Naik, Yadav Nidhi, Krishan Kumar, Sandeep Grover

**Affiliations:** Department of Psychiatry, Postgraduate Institute of Medical Education and Research, Chandigarh, India

**Keywords:** premenstrual dysphoric disorder, menstrual, validity, Robins and Guze, attention deficit hyperactive disorder, postpartum, bipolar, depression

## Abstract

The World Health Organization (WHO) and American Psychiatric Association (APA) have recognised premenstrual dysphoric disorder (PMDD) as an independent diagnostic entity, legitimising the distress and socio-occupational impairment experienced by affected women. However, the biological validity of this diagnosis remains inexplicit. This illness has also been criticised for a feminist-led, sympathetic reaction to the modern cultural challenges of urban, literate, employed, high-functioning women. This article systematically reviews existing literature on PMDD using the criteria established by Robins and Guze for the validity of a psychiatric diagnosis (clinical description, laboratory study, exclusion of other disorders, follow-up study, and family study). Despite the early recognition of premenstrual syndrome (PMS) in the 1950s, the research has encountered challenges due to two groups of proponents viewing it with psychologising bias and medicalising bias. PMDD is currently understood as the most severe form of PMS, characterised by the presence of psychological features. Recent evidence suggests that PMDD perhaps has neurodevelopmental underpinnings (attention deficit hyperactive disorder, adverse childhood experiences) affecting the fronto-limbic circuit that regulates the emotions. In addition, the affected individuals exhibit an increased sensitivity to gonadal hormonal fluctuations as observed during premenstrual, pregnancy, and perimenopausal phases of life. The prevalence is comparable between high-income countries and low- and middle-income countries (LAMIC), refuting the notion that it mostly affects modern women. Instead, a greater prevalence is observed in LAMIC. Despite the fact that educated women possess knowledge regarding the importance of getting help, there is a prevalent issue of inadequate help-seeking behaviour. This can be attributed to the perception of seeking help as an isolating experience, which is influenced by profound internalised stigma and discrimination in the workplace. Future studies must aim to develop culturally validated assessment tools and more research to understand the life course of the illness, in addition to systematically examining for more biological validators (animal models, genetics, imaging, neurotransmitters).

## 1. Introduction

The somatic, affective, and cognitive symptoms during the luteal stage of the menstrual cycle are commonly known as premenstrual symptoms. These symptoms are on a spectrum of mild-to-moderate severity that is often culturally normalised. The severe form with greater regularity interfering with daily life is defined as premenstrual syndrome (PMS). The predominant and extreme psychological form of PMS is conceptualised as premenstrual dysmorphic disorder (PMDD). In the latest Diagnostic and Statistical Manual for mental disorders—fifth edition text revision (DSM)–5-TR, PMDD is diagnosed when “*a patient, in most of her menstrual cycles during the past one year, has at least five symptoms such as affective lability, irritability, depressed mood, anxiety (at least one of these four), loss of interest, fatigue, feeling emotionally overwhelmed, and physical symptoms*” (1)*.* These symptoms must be present a week before the onset of menstrual flow and improve within a few days after, following a cyclical pattern from menarche to menopause. The symptoms must occur only during the luteal phase in most cycles during the last 1 year and include a cluster of affective, somatic, and cognitive symptoms causing significant distress, interfering with work, school, or usual social activities, and lower quality of life. These disorders are treatable—selective serotonin reuptake inhibitors (SSRIs) such as sertraline, paroxetine, fluoxetine, and escitalopram have been shown to treat both the psychiatric as well as physical symptoms (2); other medications that have shown benefit include quetiapine (3) (as an adjunct to an SSRI), oral contraceptives (4), and calcium supplementation (5). Among non-pharmacological treatments, evidence suggests that cognitive behaviour therapy may be helpful (6). In a study by Hylan et al. (7), it was estimated that women have approximately 481 menstrual cycles during their lifespan, and women with PMDD have approximately 6.4 days of severe symptoms during each menstrual cycle, spending over 3,000 days in the premenstrual phase. However, studies have found widely variable prevalence rates for PMS (8, 9) (∼4%–80%) and PMDD (10, 11) (up to 10%). Several etio-pathological theories have been proposed and found inconclusive. Given its self-remitting and cyclical nature, the efficacy of medical and psychological interventions is contentious. The validity of PMDD stands arguable among clinicians and researchers alike since the 1980s. This review used the gold standard Robins and Guze's (12) five phases of validating a psychiatric diagnosis (clinical description, laboratory study, exclusion of other disorders, follow-up study, and family study) to critically examine the published literature on PMDD for identifying important knowledge gaps and setting the research agenda to enhance the understanding of the prevalence and associated biopsychosocial factors through a neurodevelopmental lens.

### 1.1. Clinical description

Ancient medical literature described menstrually related physical and psychological problems approximately 4 millennia ago. Interestingly, proponents of either school of physical symptoms-predominant or psychological neurosis were dogmatically leading to biases.

#### 1.1.1. Psychologising bias

Kahun Gynaecological Papyrus (c.1800 BC) illustrated menstrual-related symptoms such as musculoskeletal aches, discomfort, and menorrhagia and attributed them to the females having a “womb” (13). The womb was ascribed to physical symptoms until Thomas Sydenham proposed “emotional experiences” in women as “hysteria” (suffocation of the womb) in the 17th century (13). In the late 19th century, Sigmund Freud hypothesised hysteria as a neurotic clinical entity. During this period, there was a prevailing notion that all women were pathologically emotional, causing discrimination, marginalisation, and devoid of electoral rights, among many violations (14).

#### 1.1.2. Medicalising bias

The discovery of female sex hormones in the 1950s paved the way for scientific understanding of premenstrual nervous tension. Greene and Dalton (15) studied physical symptoms and renamed it PMS. During the 14 days of the luteal phase, the progesterone levels supersede the oestrogen levels. Progesterone provides negative feedback to the anterior pituitary, initially causing a sharp fall in the levels of follicle-stimulating hormone (FSH) and luteinising hormone (LH) in the late luteal phase. At this stage, the corpus luteum regresses, leading to a sharp decrease in its production of 17-beta-oestradiol and progesterone. The rapid changes in the progesterone levels during the luteal phase of the menstrual cycle have an impact on serotonin and may result in premenstrual symptoms, despite the presence of normal ovarian function (16). Owing to this theory, it was formerly termed late luteal phase dysphoric disorder (LLPDD) and included in the Appendix A (proposed diagnostic category for further study) of DSM-IIIR in 1987 (17). Medicalising PMDD geared the research towards investigating the efficacy of progesterone and its congeners; however, these interventions have been found to be ineffective and overlooked the potential role of psychotropic and psychological interventions for a long time.

The guidelines on PMS provided by the Royal College of Obstetricians and Gynaecologists (RCOG 2016) urged the integration of biological and psychological constructs to define the illness characteristics without unintentionally pathologising the menstrual cycle or stigmatising an entire gender (18).

#### 1.1.3. Diagnostic guidelines

In DSM-IV, LLPD disorder was renamed “premenstrual dysphoric disorder” (PMDD) due to an empirical evidence indicating premenstrual onset and early follicular phase offset in the menstrual cycle and included it in Appendix III (diagnosis for further study) of DSM-IV (19). The DSM-IV work group recommended the prospective use of standardised rating instruments to determine the true prevalence of PMDD (19). The work group proposed that incorporating prospective daily ratings could improve the accuracy of diagnosis by confirming the specific timing of symptom onset and offset in relation to the menstrual phase. This approach would also help prevent the inappropriate inclusion of women experiencing milder symptoms or premenstrual worsening of existing affective disorders (19).

The American College of Obstetricians and Gynaecologists (ACOG) requires the presence of at least one affective symptom (e.g., anger outbursts, anxiety, confusion, depression, irritability, or social withdrawal) and one somatic symptom (e.g., abdominal bloating, breast tenderness or swelling, headache, joint or muscle pain, swelling of extremities, or weight gain) for a diagnosis of PMS. In contrast, the DSM-IV criteria require only the presence of somatic symptoms.

After DSM-5 recognised PMDD as an independent diagnosis, the World Health Organization (WHO) added it to the International Statistical Classification of Diseases and Related Health Problems, Eleventh Revision (ICD-11) with code GA34.41, under diseases of the genitourinary system (20). PMDD is cross-listed in the sub-grouping of depressive disorders due to the prominence of mood symptomatology. Given the debilitating nature of this illness, both traditional classificatory systems (DSM and ICD) have designated it as an independent diagnostic entity (Table 1).

**Table 1:** ICD 11 and DSM-5 criteria for PMDD.

ICD 11 (20)	DSM-5 (1)
A. During a majority of menstrual cycles within the past year, a pattern of mood, somatic, or cognitive symptoms is present that begins several days before the onset of menses, starts to improve within a few days after the onset of menses, and then becomes minimal or absent within approximately 1 week following the onset of menses. The temporal relationship of the symptoms and the luteal and menstrual phases of the cycle should ideally be confirmed by a prospective symptom diary over at least two symptomatic menstrual cycles. B. The symptoms include: • At least one affective symptom such as mood lability, irritability, depressed mood, or anxiety • Additional somatic or cognitive symptom(s) such as lethargy, joint pain, overeating, hypersomnia, breast tenderness, swelling of extremities, concentration difficulties, or forgetfulness. C. The symptoms are not better accounted for another mental disorder (e.g., a mood disorder, an anxiety, or a fear-related disorder). D. The symptoms are not a manifestation of another medical condition (e.g., endometriosis, polycystic ovary disease, adrenal system disorders, and hyperprolactinaemia) and are not due to the effects of a substance or medication on the central nervous system (e.g., hormone treatment, alcohol), including withdrawal effects (e.g., from stimulants). E. The symptoms result in significant distress or significant impairment in personal, family, social, educational, occupational, or other important areas of functioning. Boundary with normality (threshold) Mild mood changes (e.g., increased emotional lability, irritability, subjective tension) that occur during the late luteal or menstrual phase of the cycle for many women should not be labelled as PMDD. In contrast to PMDD, these symptoms are less intense and do not typically result in significant distress or impairment.	A. In the majority of menstrual cycles, at least five symptoms must be present in the final week before the onset of menses, start to improve within a few days after the onset of menses, and become minimal or absent in the week post-menses. B. One (or more) of the following symptoms must be present: 1. Marked affective lability (e.g., mood swings; feeling suddenly sad or tearful, or increased sensitivity to rejection). 2. Marked irritability or anger or increased interpersonal conflicts. 3. Marked depressed mood, feelings of hopelessness, or self-deprecating thoughts. 4. Marked anxiety, tension, and/or feelings of being keyed up or on edge. C. One (or more) of the following symptoms must additionally be present, to reach a total of five symptoms when combined with symptoms from Criterion B above. 1. Decreased interest in usual activities (e.g., work, school, friends, hobbies). 2. Subjective difficulty in concentration. 3. Lethargy, easy fatigability, or marked lack of energy. 4. Marked change in appetite; overeating; or specific food cravings. 5. Hypersomnia or insomnia. 6. A sense of being overwhelmed or out of control. 7. Physical symptoms such as breast tenderness or swelling, joint or muscle pain, a sensation of “bloating,” or weight gain. Note: The symptoms in Criteria A–C must have been met for most menstrual cycles that occurred in the preceding year.
	D. The symptoms cause clinically significant distress or interference with work, school, usual social activities, or relationships with others (e.g., avoidance of social activities; decreased productivity and efficiency at work, school, or home). E. The disturbance is not merely an exacerbation of the symptoms of another disorder, such as major depressive disorder, panic disorder, persistent depressive disorder, or a personality disorder (although it may co-occur with any of these disorders). F. Criterion A should be confirmed by prospective daily ratings during at least two symptomatic cycles. (Note: The diagnosis may be made provisionally prior to this confirmation.) G. The symptoms are not attributable to the physiological effects of a substance (e.g., a drug of abuse, a medication).

#### 1.1.4. Assessment tools

The screening tools for premenstrual symptoms, including both adult and adolescent versions, are widely used in clinical practice (21, 22). The structured clinical interview for DSM-IV-TR PMDD (SCID-PMDD) is a diagnostic interview schedule developed in 2013 (23), which includes five scales (24–28) for self-monitoring of PMDD during the prospective daily ratings over at least two menstrual cycles. Among them, the Daily Record of Severity of Problems (DRSP) based on the DSM-IV criteria for PMDD is the most commonly used (24). Carolina Premenstrual Assessment Scoring System (C-PASS) is based on the DSM-5 criteria for PMDD in four diagnostic dimensions (symptoms, severity, cyclicity, and chronicity). The C-PASS assessment tool is sensitive to predict sub-threshold PMDD, i.e., women with a menstrual-related mood disorder (MRMD) who experience distress and impairment sufficient to warrant treatment but do not meet the full DSM-5 criteria for PMDD (28). They have high internal consistency of 0.8–0.9 (Table 2). The ICD-11 provided guidance on establishing the boundary with normality for the exclusion of mild premenstrual mood changes; nevertheless, culturally-adapted and standardised tools are yet to be developed.

**Table 2:** Assessment tools for PMDD.

Authors	Assessment tool	Description	Psychometric properties	Remarks
Screening instruments				
Steiner et al. (2003) (21)	PSST [(premenstrual symptoms screening tool (21)]	Based on the DSM-IV criteria of PMDD. Contains two sections and 19 questions. Classified into “mild/no PMS,” “moderate to severe PMS,” and “PMDD”	Sensitivity coefficients—0.9 Specificity coefficients—0.77 Cronbach's alpha for internal consistency—0.91 Test–retest reliability—0.56	Useful for screening, but not for the diagnosis or monitoring of severity Widely used in many studies worldwide Cultural validation is available in Brazil, Iran, and Italy
Steiner et al. (2011) (22)	PSST—Adolescent version (22)	Adopted from PSST	Not available	Widely used in adolescents
Diagnostic instrument				
DSM-IV (23)	Structured Clinical Interview for DSM-IV-TR defined PMDD (23) (SCID-PMDD)	DSM-5 based semi-structured interview guide	No reliability or validity data is available	Developed in 2013
Severity rating scales				
Steiner et al. (1980) (27)	Premenstrual Tension Syndrome Rating Scale self-report and an observer version (27)	For diagnosis of PMS and PMDD, and monitoring symptoms severity through prospective daily rating (for Self-Rating version)	Internal consistency (alpha = 0.89–0.93)	The self-rating version contains 36 dichotomous items (Yes or No) on PMS/PMDD symptoms
Steiner and Steiner (1999) (26)	Visual Analog Scale (VAS)	Used to rate each of the four core symptoms of PMDD: mood swings, irritability, tension, and depression	Cronbach's alpha > 0.90 for Internal consistency	Measures subjective perception of PMDD
Feuerstein and Shaw (25)	Calendar of Premenstrual Experiences (25) (COPE)	Includes 22 symptoms grouped into four categories: mood reactivity, autonomic/cognitive, appetitive, and related to fluid retention	Internal consistency (alpha) (0.93–0.94)	Self-monitoring diary for the prospective ratings of PMDD
Endicott et al. (2005) (24)	Daily Record of Severity of Problems (24) (DRSP)	Assessment of the DSM-IV criteria for PMDD and to assess severity of symptoms and impairment. It is recorded daily by the subject throughout her two menstrual cycles	Internal consistency (*α* = 0.91–0.96)	Most commonly used. It can be downloaded online and used with permission from the original author
Eisenlohr-Moul et al. (28)	Carolina Premenstrual Assessment Scoring System (28) (C-PASS)	Computerised version of DRSP	98% accuracy to diagnose PMDD	C-PASS is only validated to diagnose DSM-5 PMDD

#### 1.1.5. Prevalence of PMDD

The majority of global prevalence studies have predominantly focused on PMS since the 1950s. There has been a limited number of country-wide prevalence studies conducted on PMDD in the last two decades only, and most of these studies are cross-sectional observations (Table 3). Ten out of 14 cross-sectional studies are conducted among adolescents and young women (9, 29–37), and only five studies examined the prevalence in middle-aged women (38–42). A cross-sectional study found a prevalence rate ranging from 2.2% to 3.7% across all reproductive-age groups and a greater incidence rate in women of age 45–54 years, indicating that PMDD is a disorder spanning from menarche to menopause (41).

**Table 3:** Prevalence of PMDD.

Author, year, country	Sample characteristics	Age (years) Mean (SD)/range	Study design	Tools	Findings
CROSS-SECTIONAL STUDIES					
Low- and middle-income countries					
Asia					
General population					
Qiao et al., China (40)	4,715 Population-based study	18–45	PMS/PMDD	ACOG recommendation, DRSP for 2 months	PMDD—2.1% PMS—21.1%
Pal et al., Pakistan (40)	402 Population-based study at three cities	15–49	PMDD	Checklist of 23 premenstrual symptoms	ICD 10%–79.9% ACOG—12.7% DSM-IV—5.5%
Specific group—students					
Dutta and Sharma, India (38)	8,542 girls and women	10–50	PMS/PMDD Meta-analysis of cross-sectional studies, case-control studies or cohort studies	Penn State Daily Symptom Report; PMS self-Evaluation Questionnaire; PSST—adult and adolescent versions. SCID-PMDD; Shortened Premenstrual Assessment Form; Self-screening quiz for PMDD as per the DSM-IV-TR criteria.	Pooled prevalence PMDD 8% (95% CI: 0.60–0.10) PMS 43% (95% CI: 0.35–0.50) High heterogeneity observed.
Thakrar et al., India (30)	661 Medical paramedical students	19.5 ± 1.5	PMDD	PSST DRSP	5.04% students screened positive by PSST and prevalence of PMDD was 4.43% by DRSP
Pattanashetty et al., India (33)	900 High school students	12–16	PMDD	Pre-tested, Semi-structured questionnaire	4.89%
Koganti et al., India (35)	1,800 Medical Students	18–25	PMDD	Penn's daily symptom rating scale (self-administered for 2 months) and an interview based on diagnostic and statistical manual—5 diagnostic criteria	11.11%
Durairaj and Ramamurthi, India (9)	1,112 College students	17–25	PMS/PMDD	PSST	Prevalence of moderate to severe PMS was 14.3% and PMDD was 3.7%
Bansal et al., India (36)	592 College students	21.1 ± 2.6	PMDD	PSST	The prevalence of PMS/PMDD was 46.1%, out of which 10.2% met the criteria for PMDD
Shehadeh and Hamdan-Mansour, Jordan (37)	858 Students	22.8± 0.3	PMDD	DRSP based on DSM-IV	Prevalence of PMDD was 7.7%
Africa					
Specific group—students					
Duko et al., Ethiopia (29)	4,993 High school/College university students	Not specified	PMDD Meta-analysis of cross-sectional studies	PSST DSM-IV DSM-5 ACOG Self-administered questionnaire	Pooled prevalence—54.5% (95% CI 40.8–67.6)
Eldeeb et al., Egypt (31)	755 students	21.2 ± 3.7	PMDD Cross-sectional study	Questionnaires covering Diagnostic and Statistical Manual of Disorders (DSM-5) criteria to diagnose PMDD	21.1%
High-income countries					
Clinical population					
Ogebe et al. (32) three centres, midwestern United States and two Nigerian cities	537 Clinical population	13–21	PMDD Cross-sectional survey	Modified version of the PSST	Overall prevalence—4.1% (Maiduguri, Nigeria 6.5%; Lagos, Nigeria 3.1%; Akron, United States—2.9%). No statistically significant difference at the three centres
General population					
Tschudin et al., Switzerland (41)	3,522 Population-based Health Survey	15–54	PMS/PMDD	PSST	Prevalence of PMDD with age group 15–24: 3.0% 25–34: 2.2% 35–44: 3.5% 45–54: 3.7%
Dueñas et al., Spain (42)	2,108 Cross-sectional population-based survey	15–49	PMS/PMSS	PSST	PMS 73.7% PMDD 1.1%
LONGITUDINAL STUDIES					
General population					
Wittchen et al. (43)	1,488	14–24	Prospective longitudinal community survey Follow-up: 48 months	Diagnostic assessments—Composite International Diagnostic Interview (CIDI) and 12-month PMDD diagnostic module administered by clinical interviewers	Baseline 12-month prevalence DSM-IV PMDD 5·8% PMDD syndrome stable across 48 months, <10% complete remissions among baseline PMDD cases

There is a prevailing belief that PMDD is a disorder mostly observed in developed, high-income countries and criticised as a cultural syndrome of urbanisation. Contrary to that, Ogebe et al. (32) noticed greater reports of PMDD in two Nigerian cities as opposed to the United States. Studies conducted in high-income countries reported the prevalence rate of PMDD to vary from 3% to 4.1%, while the prevalence rate of PMDD in low- and middle-income countries (LAMIC) ranged from 3.7% to 11%. The greater prevalence observed in LAMIC indicates the need to thoroughly examine the socio-economic determinants of PMDD, such as literacy, economic decline, migration, public health policies, and laws protecting women against violence and discrimination.

In the general population, the prevalence rate of PMDD ranges from 2.1% to 79.9%, depending on the assessment tools (39, 40). In a specific group (student population), it was found that the prevalence rate ranges from 3.7% to 10.2% (9, 36), while in the clinical population, it ranges from 2.9% to 4.1% (32).

### 1.2. Laboratory studies

Neurotransmitter levels and hormonal changes are extensively studied in PMS but are limited for PMDD. Tryptophan challenge and tyrosine depletion tests concluded lower serotonin, dopamine, and norepinephrine levels during the luteal phase (44). However, the severity of PMS was correlated with the depletion of tryptophan only, implicating a hypo-serotonergic theory and the potential therapeutic role of SSRIs in PMS and PMDD. PMDD symptoms were not associated with oestrogen and progesterone levels (45). Allopregnanolone is a neurosteroid and an anxiolytic metabolite of progesterone that acts at the GABA-A receptor. Lower baseline allopregnanolone and a more marked increase in allopregnanolone levels were reported in women with PMDD after administering progesterone. However, no similar changes were observed in women with depression or in healthy subjects. The rapid efficacy of SSRIs in PMDD has been attributed to their ability to increase allopregnanolone levels in the brain, enhancing GABA-A receptor function and alleviating anxiety (46). Single nucleotide polymorphisms (SNPs) in the serotonergic 5HT1A receptor (47) and oestrogen receptor *α*-gene (ESR1) (48) have been found to be associated with PMDD. Met-allele carriers of brain-derived neurotropic factor (BDNF) (Val66Met SNP) had shown impaired fronto-cingulate cortex activation during the luteal phase (49).

There are no major structural brain changes in women with PMDD (Table 4). Functional imaging studies have reported increased amygdalar activity in the limbic region and decreased activity in prefrontal cortical structures, such as the anterior cingulate cortex (ACC), medial prefrontal cortex (mPFC), and dorsolateral prefrontal cortex (DLPFC), more pronounced during the late luteal phase. These findings are inconsistent. The reversal of hypo-reactivity in the DLPFC during the follicular phase implies that the prefrontal hypoactivity is transitory and excessive during the late luteal phase, which requires replication studies (50). White matter integrity has not yet been studied. Although patients with PMDD have higher cerebellar grey matter volume and metabolism as well as altered serotonergic and GABAergic neurotransmission, it is better distinguished by differentiating amygdalar and fronto-cortical function in response to emotional stimuli. There is a need for further structural, chemical, and functional brain signatures in order to gain a comprehensive understanding of the complex, emotional, behavioural, physical, and cognitive symptoms. Currently, they are understudied and inconsistent.

**Table 4:** Summary of laboratory evidence for PMDD.

Authors	Study sample	Age (years) Mean (SD)/range	Findings
Genetics			
Comasco (49)	PMDD—31 Healthy controls—31	Not available	5-HTTLPR and BDNF Val66Met polymorphisms not associated with PMDD. Met-allele carrier -lower emotion-induced fronto-cingulate cortex activation during luteal phase in PMDD
Dhingra (47)	PMDD—53 women Healthy controls—51	PMDD—27–46 Healthy controls—22–48	Presence of at least one C allele of serotonergic 5HT1A receptor associated with a 2.5-fold increased risk of PMDD
Huo (48)	PMDD—91 Healthy controls—56	39.5 ± 5.9	SNPs in oestrogen receptor α-gene (ESR1) positively associated with PMDD
Endocrinal			
Progesterone/allopregnanolone/oestrogen			
Klatzkin (51)	Prior depression with PMDD (PMDD-Dep) = 13 Non-PMDD Depression (Non-PMDD-Dep) = 12 Non-Dep PMDD = 23 Non-PMDD, Non-Dep = 29	PMDD-Dep—33.8 (1.8) Non-PMDD-Dep—36.5 (1.9) non-Dep PMDD—31.7 (2.1) Non-PMDD, Non-Dep—33.6 (1.6)	Non-Dep PMDD had higher pre-progesterone and allopregnanolone levels following progesterone administration than other groups
Hsiao (45)	PMDD = 43	30.79 (7.13)	No statistically significant correlations between depression or anxiety ratings and oestrogen or progesterone concentrations
Neurotransmitters			
Serotonin			
Rasgon (44)	PMS = 5 Healthy Control = 5	PMS—24 ± 0 Healthy control—27 ± 4	L-tryptophan challenge PMS >HC blunted whole blood serotonin response in the luteal phase
Rapkin (52)	PMS = 14 Healthy controls = 13	Age: NA	Serotonin-PMS < Healthy Control
Dopamine and norepinephrine			
Menkes (53)	PMS = 16	37.9 ± 5.8	Significant premenstrual tyrosine decrement
Neuroimaging			
Structural imaging			
Syan et al. (54)	PMDD—20 Healthy controls—25	PMDD—31.80 ± 7.33 Healthy controls—27.44 ± 7.74	No significant group effect
Berman et al. (55)	PMDD—12 Healthy controls—13	PMDD—30.9 ± 6.63 Healthy controls—29.2 ± 6.50	PMDD > HC: ↑ grey matter volume cerebellum
Protopopescu et al. (56)	PMDD—10 Healthy controls—11	29 (22–35)	No significant group effect
Functional imaging			
Emotional stimuli-reactivity			
Petersen et al. (50)	PMDD—18 Healthy controls—18	PMDD—29.2 ± 7.24 Healthy controls—25.4 ± 6.99	DLPFC reactivity for PMDD lower in the late luteal phase compared with healthy subjects and follicular phase
Gingnell et al. (57)	PMDD—14 Healthy controls—13	PMDD—35 ± 8.9 Healthy controls—33.1 ± 7.8	↑amygdala activity and functional connectivity between the amygdala, insula, and anterior cingulate cortex (ACC) during the late luteal phase in PMDD
Comasco et al. (49)	PMDD—16 Healthy controls—15	PMDD—33.3 ± 8.9 Healthy controls—30.5 ± 8.1	PMDD >HC- ↑activations in the inferior and middle frontal gyri, right superior parietal gyrus, and left angular gyrus to negative facial expressions
Gingnell et al. (58)	PMDD—14 Healthy controls—14	PMDD—35 ± 8.9 Healthy controls—32.7 ± 7.7	Luteal phase PMDD >HC hyperactivations in lateral OFC, mPFC, and DLPFC regions during the anticipation of negative pictures
Gingnell et al. (59)	PMDD—14 Healthy controls—15	PMDD—34.9 ± 8.9 Healthy controls—33.7 ± 8.4	PMDD >CS mid-follicular phase, positive correlation between P4 levels and amygdala BOLD response to angry and fearful faces, PMDD >CS: ↑ amygdala BOLD signal during the mid-follicular phase
Protopopescu et al. (56)	PMDD—8 Healthy controls—12	PMDD—27.4 (22–33) Healthy controls—28.0 (22–35)	PMDD >HC ↑ amygdala BOLD signal during the late luteal phase, ↓ NAcc activation for the positive condition in women with PMDD compared with healthy Controls during the late luteal phase.
Neurocognitive tasks			
Baller et al. (60)	PMDD—14 Healthy controls—14	PMDD—38.1 ± 8.2 Healthy controls—36.0 ± 8	PMDD > HC: ↑ activations of superior and middle frontal gyri, inferior parietal lobule and cerebellum during N-back working memory task
Bannbers et al. (61)	PMDD—14 Healthy controls—13	PMDD—34.9 ± 8.9 Healthy controls—34.9 ± 8.6	↓ activation pre- and post-central gyri, parietal cortex, right caudate nucleus, and the left insula during the mid-follicular phase PMDD >HC: ↑ left insula reactivity during response inhibition during the late luteal phase
Magnetic resonance spectroscopy			
Liu et al. (62)	PMDD—20 Healthy controls—20	PMDD—23.0 ± 1.6 Healthy controls—23.6 ± 1.4	PMDD <HC: ↓GABA = anterior cingulate cortex/medical prefrontal Cortex, basal ganglia ↓GABA/creatine ratio anterior cingulate cortex/medical prefrontal cortex, basal ganglia ↑ glutamate–glutamine/GABA ratio anterior cingulate cortex/medical prefrontal cortex, basal ganglia
Batra et al. (63)	PMDD—12 Healthy controls—13	PMDD—35.0 ± 4.61 Healthy controls—30.0 ± 8.14	Mid-FP >late LP: ↑ glutamate/creatine ratio mPFC in both groups No group effect
Epperson et al. (64)	PMDD—9 Healthy controls—14	PMDD—34.6 ± 4.5 Healthy controls—30.1 ± 6.23	PMDD <CS during Follicular Phase FP >mid/late LP: ↑ GABA in CS FP <mid/late LP: ↓ GABA in PMDD
Rasgon et al. (65)	PMDD—5 Healthy controls—7	PMDD—29.0 ± 4.5 Healthy controls—28.0 ± 9.9	mid-FP > late LP: ↓ NAA/Cr ratio mPFC GM in both groups mid-FP >late LP: ↑ Ch/Cr ratio parietal WM in both groups
Jovanovic et al. (66)	PMDD—5 Healthy controls—5	PMDD—32.4 ± 6.2 Healthy controls—30.2 ± 7.6	CS: late LP > FP: ↑ 5-HT1A binding in dorsal RN PMDD: no change

#### 1.2.1. Risk factors and protective factors

In the absence of consistent laboratory markers, a few factors were identified to pose a greater risk for PMDD such as prior traumatic events, a history of mental disorders, peripartum depression, obesity, smoking, alcohol use, and heavy drinking (67–76) (Table 5). There is little research regarding protective variables, and factors such as caffeine intake and oral contraceptive pills are still inconsistently discussed (4, 77–79).

**Table 5:** Risk factors and protective factors for PMDD.

**Risk factors**	**Protective factors**
Past traumatic events (67, 68)	Oral contraceptives (4, 79) (inconsistent)
Cigarette smoking (RR of 1.93 for 20 pack-years) (70)	
Obesity RR in women with a BMI of 35.0 kg/m^2^ was 1.66 (71)	
Family history of PMS and PMDD (72, 73)	
History of postpartum depression (74) Major depression past (75, 76)	
Alcohol intake (76) and caffeine consumption (77, 78) (inconsistent)	

#### 1.2.2. Link to postpartum depression

Oestrogen and progesterone affect a variety of biological processes, brain networks, and mood-related behaviours; therefore, alterations in their levels may result in depressive symptoms. Women who are sensitive to hormonal changes may experience both PMS and postpartum depression (PPD) due to the sudden reduction in hormone levels that occurs during the luteal period as well as after delivery. The prevalence of PMDD symptoms was shown to be significantly higher in women with PPD compared with those without PPD, with a medium effect size (80). A meta-analysis of seven retrospective studies found a strong positive association between PMDD and PPD (81). A prospective cohort study found that higher severity of depressive symptoms in the first month following childbirth significantly predicted the incidence of PMDD during the first year of the postpartum period, implicating that PPD can be a risk factor for PMDD. However, replication studies are needed to substantiate this finding.

#### 1.2.3. Link to climacteric phase

Women with a PMDD history exhibit significantly more severe depressive features than those without PMDD during their perimenopausal phase (80). It indicates that PMDD has a trajectory to develop into climacteric depression in women. However, longitudinal studies are required to investigate this matter.

#### 1.2.4. Link to personality disorders

Women with PMDD exhibit less compulsive, rather more passive/aggressive, borderline/cycloid, and depressive and manic symptoms (82). One study found them to have higher obsessional personality features in the absence of a definitive diagnosis of a personality disorder (83). Another study found a higher risk of avoidant personality disorder, but only in women who are aged 30 years or older (84). Ducasse et al. (85) found that independent of the time of the menstrual cycle, women with PMS or PMDD have an impulsive-aggressive personality style. The association between trait anger and both PMS and PMDD was observed to be independent of all other personality traits. A higher level of anger is considered to pose a higher risk of experiencing both PMS and PMDD.

#### 1.2.5. Suicidal risk

Women with PMDD and PMS are at seven times the odds of suicide attempt and almost four times as likely to exhibit suicidal ideation compared with women without premenstrual disturbances (86). A routine assessment of suicide risk for women experiencing moderate-to-severe premenstrual disturbances is warranted, and psychosocial treatments targeting suicidality must be provided to improve their wellbeing.

### 1.3. Exclusion of other disorders

The broad presentation of PMDD frequently includes co-occurring physical symptoms (87). It is important to investigate for any abnormalities of thyroid, gynaecological problems, and anaemia that can cause physical and psychological symptoms as observed in PMDD.

When the menstrual cycle coincides with the periodicity of epilepsy, the exacerbation is known as catamenial epilepsy (88). It is seen in 10%–70% of reproductive-age women with both focal and generalised epilepsy (89). The aberrant interaction between ovarian hormones and the central nervous system (CNS) has been proposed as a potential mechanism linking menstrual cycle-related disorders such as catamenial epilepsy and PMDD (90). Nowosielski et al. (87) found that women with PMS experience a twofold increased risk of sexual dissatisfaction and increased sexual pain when compared with women without PMS. More studies are required to examine the prevalence and patterns of various sexual dysfunctions associated with PMDD.

Criterion C of ICD-11 and criterion E of DSM-5 TR state that the disturbance should not be a mere exacerbation of the symptoms of another mood or anxiety disorder. Due to inadequate awareness of PMDD, most patients present, during the symptomatic phase of comorbid depressive or anxiety illness, with a history of mood symptoms worsening during the premenstrual phase with a potential retrospective falsification coloured with dysphoric mood. In such cases, a prospective observation is the only prudent way to ascertain PMDD. The clinicians must wait for the remission of symptoms and examine the daily subjective record of PMDD symptoms to identify premenstrual worsening during the two consecutive months of a remitted phase of comorbid illness. The major distinguishing symptoms of PMDD are irritability and affect lability rather than a low mood or anxiety. Serotonin reuptake inhibitors exhibit a different profile in PMDD, including a short onset of action, thus implying that this effect is possibly mediated by different serotonergic synapses from those that are involved in the antidepressant and anti-anxiety activities of these medications (91).

In attention deficit hyperkinetic disorder (ADHD), emotional dysregulation with premenstrual worsening has been recognised as a diagnostic criterion (DSM-5TR), an overlapping feature with PMDD. Dorani et al. (80) found that 45.5% of women with ADHD have a diagnosis of PMDD.

Wittchen et al. (43) concluded that women with PMDD had an eightfold increase in the risk of bipolar disorder (BD). A study titled Systematic Treatment Enhancement Program for Bipolar Disorder (STEP-BD) found that women with comorbid PMDD experienced a worse course of illness in the form of an earlier age at the onset for BD, increased number of episodes, more severe mood symptoms in the perinatal period, higher comorbidities (anxiety spectrum, ADHD, and substance use disorders), and higher rates of rapid cycling (92). A systematic review of 17 studies concluded that women with PMS or PMDD had more frequent diagnoses of BD-I or BD-II than those without PMS or PMDD. Women with BD-II and cyclothymia are more commonly diagnosed with PMS or PMDD. Women with both BD and PMS were found to have increased severity of manic symptoms, particularly irritability, anger, lability of mood, and sleep deprivation. This suggests that PMDD may induce or perpetuate mania in individuals with BD. In addition, a worse therapeutic response and more frequent relapses were observed in BD patients with comorbid PMDD (93). These alarming findings insinuate that PMDD and BD might have shared pathophysiological processes.

### 1.4. Follow-up study

To date, one longitudinal study (43) (Table 2) conducted over 48 months of the prospective investigation reported that only 10% of baseline PMDD patients have remission of symptoms, and the rest continued to have features of PMDD, suggesting the stability of diagnosis.

### 1.5. Family study

Genetic vulnerabilities are indicated through family research. Studies on families, notably those involving twins, point to connected heritable factors. Table 6 (94–98) summarises the findings from several studies on the heritability of PMS using twin samples. There is no family study available regarding PMDD, and its heritability is unknown. PMS family studies collectively suggest that PMS has a strong genetic component, with higher proband-wise concordance in monozygotic twins compared with dizygotic twins (94, 97). Additive genetic influences were identified, and a genetic correlation was found between PMS and neuroticism and lifetime major depression (95).

**Table 6:** Summary of twin studies on PMS/PMDD.

**Authors**	**Study subjects**	**Concordance rates**	**Genetic heritability**
Jahanfar et al. (94)	193 subjects [inclusive of same gender twins (*n* = 176) and females from opposite sex twin sets (*n* = 17)]	43.0% in monozygotic and 46.8% in dizygotic twins. Proband-wise concordance for PMS was higher in monozygotic (0.81) than in dizygotic twins (0.67), indicating a strong genetic effect	Quantitative genetic modelling found that a model comprising of additive genetic (A) and unique environment (E) factors provided the best fit of A: 95%, E: 5% suggesting 95% genetic heritability
Treloar et al. (95)	720 female twin pairs (454 monozygotic and 266 dizygotic) Australian National Health and Medical Research Council Twin Register	Genetic correlations of 0.62 between reported PMS and neuroticism, and 0.70 with lifetime major depression, 39% of the genetic variance of PMS was not explained by these factors	Indicating weaker genetic effect
Kendler et al. (72)	Virginia Twin Registry two assessments, 72 h apart 314 monozygotic and 181 dizygotic twin pairs	Stability of psychological symptoms of PMS.	A best-fitting twin-measurement model estimated the heritability of the stable component of premenstrual symptoms at 56% and showed no impact of family environment factors
Dalton et al. (97)	31 pairs of twins Prospectively examined premenstrual symptoms	Significantly higher concordance rate in monozygotic pairs (93%, 14 of 15) than in dizygotic pairs (44%, 7 of 16)	Indicating a strong genetic effect
Condon et al. (98)	157 monozygotic and 143 dizygotic female twin pairs Self-report questionnaire on premenstrual syndrome (PMS)	Correlation in global PMS scores nearly twice as great for monozygotic twins (*r* = 0.55) as for dizygotic (*r* = 0.28) pairs	Indicating a strong genetic effect

### 1.6. Is PMDD a neurodevelopmental disorder?

**Figure 1:**
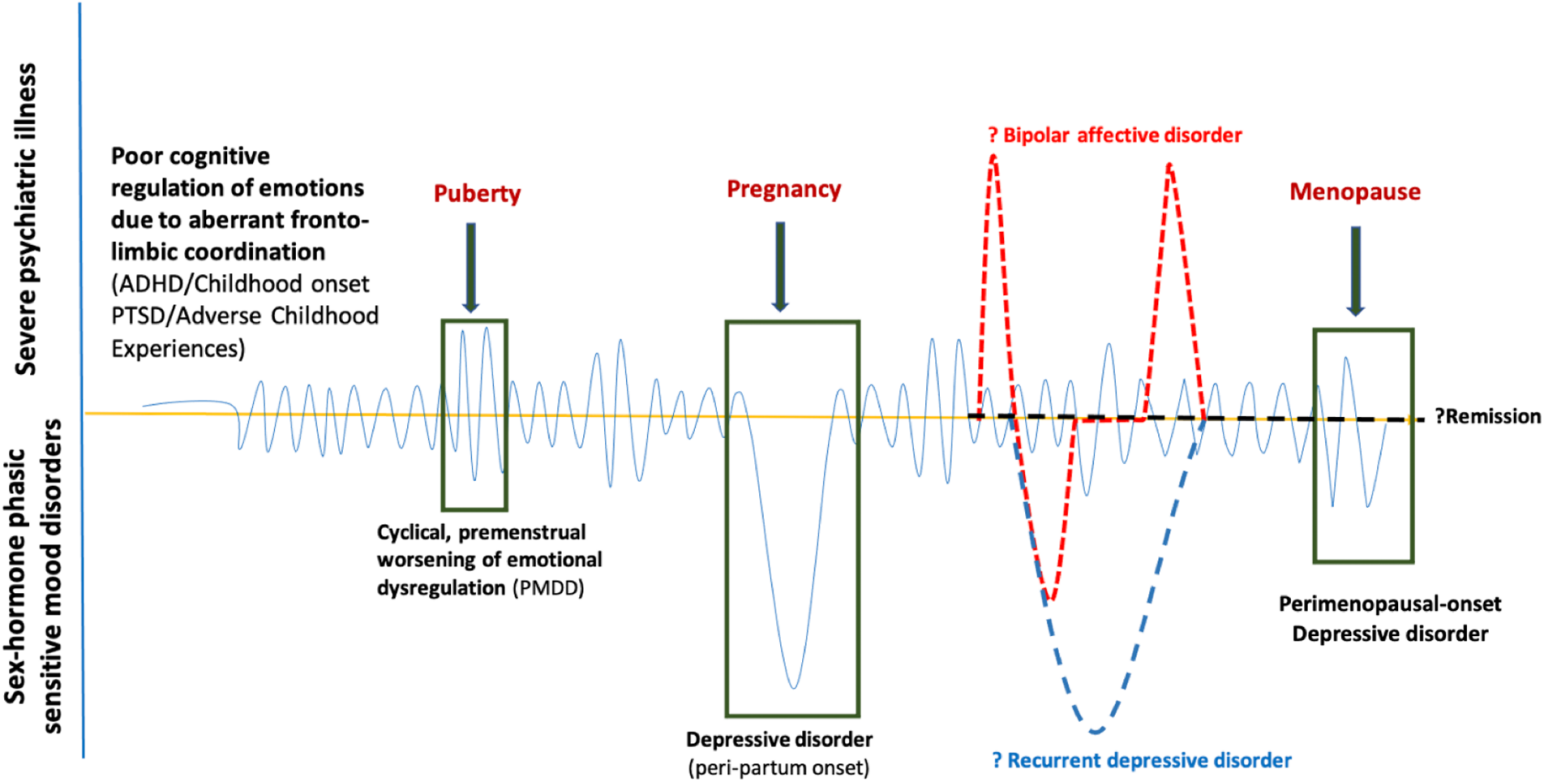
Putative lifetime trajectories of PMDD.

Dorani et al. (80) found that the prevalence of PMDD, PPD, and climacteric mood symptoms (Cohen's d: 3.71) were high in women with ADHD compared with the general population. ADHD may be an early risk factor for the development of PMDD and BD (99), with a shared neurobiological and genetic underpinning. “Fronto-limbic disconnection” can be hypothesised to understand the continuum of childhood-onset dysfunctional emotional brain networks (100), either genetic (ADHD) or acquired (traumatic or adverse childhood experiences), expressed as poorer emotion regulation with depressive, anxiety, and behavioural features of PMDD during the gonadal hormonal rapid fluctuations phases (luteal, pregnancy and perimenopausal) (Figure 1). It is yet unknown how much this childhood-onset dysfunctional emotional brain network remains as a personality trait marker or elevates the risk for bipolar disorder, recurrent depressive disorder, or remits in due course, reiterating the need for more longitudinal studies to understand the origin and evolution of the disease process in the developing brain in children at-risk for PMDD.

## 2. Discussion

The recognition of PMDD has gone far beyond the debate of whether it is an idiom of distress or a cultural syndrome. Through the application of Robins and Guze's diagnostic validation exercise, we found that the diagnostic guidelines yielded diagnostic stability (12). The validated screening and diagnostic assessment tools demonstrate favourable psychometric properties and require more cultural adaptations. The laboratory findings require replication studies. More follow-up studies and family studies of PMDD are needed. Despite the shortcomings of not having scientifically robust biological validators, as of today, it is a psychobiological illness similar to depressive disorder but has a cyclical course warranting the need for animal models, genetic risks, changes in neurotransmitters and neural network activity during the active symptomatic and remitted phases. PMDD is a disabling disorder as it reduces the work efficiency of women during their most productive years of life. There is evidence suggesting that it might precede peripartum and perimenopausal depressive disorders. Therefore, obstetricians, gynaecologists, and mental health professionals must closely monitor and intervene to prevent or alleviate subsequent depressive episodes. Accurate assessment and diagnosis of PMDD require rigorous training of community workers such as midwives and nurses and primary care physicians. The extent to which individuals seek assistance for PMDD varies worldwide due to factors such as age, subjective perception, and retrospective vs. prospective reporting (retrospective reporting is more prone to false positives and overdiagnosis, whereas a minimum of 2 months of prospective daily ratings is more associated with drop-out from clinical consultations), cultural context, awareness of the illness, and internalised stigma.

Only one 4-year longitudinal study found that only 5% of PMDD women had remission, while the remaining participants continued to suffer from PMDD symptoms with 4.4 odds of increased suicidal attempts, 7.3 odds of having more than three comorbidities, and the highest odds of 8.1 for bipolar disorder II (43). Women with ADHD had an elevated prevalence of hormone-related mood symptoms PMDD, PPD, and perimenopausal mood symptoms throughout their lives. It is suggestive of a plausibly aberrant cognitive control of mood developmentally. We generated a neurodevelopmental hypothesis based on observational/descriptive studies, and there are no biological studies conducted yet to support this hypothesis.

More research must probe into understanding the life course of PMDD, aiming to identify and intervene in the early stages, optimistically when someone is at-risk (early presentation of ADHD, adverse childhood experiences, initial traumatic experiences) to mitigate the duration, severity, and frequency of PMDD episodes.

Recent evidence suggests the crucial role of oestradiol and progesterone in modulating the neuronal network activity associated with emotion processing and attention and reward functions in susceptible women by regulating the synthesis of important neurotransmitters such as serotonin, noradrenaline, dopamine, glutamate, and GABA (101). Despite the suggestive role of gonadal hormones in PMDD, the clinical trials found limited efficacy of oral contraceptive pills (containing oestrogen and progesterone/drospirenone) confounded with high placebo rates (102), thus identified as the second-line drug for PMDD (103). This suggests that gonadal hormones may not have a direct implication in the development of PMDD, but rather they could potentially be involved through other biological mechanisms. The emergence of trials involving SSRIs or serotonin–norepinephrine reuptake inhibitors (SNRIs) can be attributed to the influence of gonadal hormones on serotonergic and norepinephrine changes. Serotonergic drugs were found to be modestly efficacious, with daily or intermittent dosing and minimal adverse effects (104). SSRI administration exclusively during the luteal phase may be a more effective treatment option, considering its self-remitting nature. Either continuous or luteal phase-only, SSRI administration has been regarded as the first line of treatment for PMDD (103). Psychological treatments such as cognitive-behavioural intervention are more efficacious in reducing the mood and behavioural symptoms of PMDD when compared with SSRI, while the latter was more efficacious in reducing the physical symptoms of PMDD (105). Emotion-focused group therapy (EFGT) with components of strengthening emotion regulation skills, increasing positive interactions, and breaking down negative cycles of interaction had been studied for PMDD women. EFGT was found to improve self-compassion and sexual function and reduce the components of pain perception and couple burnout (106). There are a variety of alternative and complementary medicine treatments under evaluation such as nutraceuticals, acupuncture, and yoga, *Vitex agnus-castus* (107)*, Hypericum perforatum* (108, 109), *Crocus sativus* (110), *Elsholtzia splendens*, and *Ginkgo biloba* (111, 112). Neuromodulation techniques can be explored to strengthen the connectivity between prefrontal control and limbic structures to improve the symptoms and prevent future episodes.

Current diagnostic classificatory systems have rightfully acknowledged the glaringly high prevalence, distress, and dysfunction associated with PMDD. While prevalence studies reported the presence of PMDD in women from menarche to menopause, no study has mentioned regarding the typical age at the onset of illness. Furthermore, the naturalistic course and outcome of PMDD symptoms are unclear from the existing literature. This review highlighted the paucity of observational studies to understand the life trajectories of PMDD that have been limiting the clinician's judgment in diagnosing the illness and posing a dilemma on what to expect for the future course of PMDD and appropriate treatment duration. This review might resolve the clinician's dilemma of diagnosing the illness with the recommended 2 months of prospective daily symptom ratings. PMDD is a unique diagnostic entity, neither a variant of depression nor an anxiety disorder. Besides the biological validators of each symptom and syndrome-level PMDD, more research on ecological validators of PMDD is required to delineate it from underlying personality traits and acute cyclical psychological reaction to any physical stress.
